# Farewell to Prof. Winfried Kahlke, born December 30, 1932, died May 1, 2025

**DOI:** 10.3205/zma001808

**Published:** 2026-02-17

**Authors:** Birgit Wulff

**Affiliations:** 1 Ärztekammer Hamburg, Hamburg, Germany

## Prof. Winfried Kahlke (30.12.1932-01.05.2025)

On May 1, Professor Emeritus Dr. Winfried Kahlke, former chair of higher education didactics at the University Medical Center Hamburg-Eppendorf (UKE), passed away after a long illness at the age of 92.

Born on December 30, 1932, in Brokstedt, Schleswig-Holstein, Winfried Kahlke studied medicine in Kiel and Heidelberg. He was licensed to practice medicine in 1961 and subsequently worked as a research assistant at the Physiological-Chemical Institute of the University of Cologne. In 1971, he completed his habilitation at the University of Heidelberg after identifying phytanic acid as the substrate responsible for Refsum’s disease, a metabolic disorder causing severe neurological deficits. In 1972, he became a specialist in internal medicine, and in 1974 he accepted the newly established professorship for higher education didactics in medicine at the University Medical Center Hamburg-Eppendorf.

His then-novel ideas on reforming medical education have long since become part of everyday academic life: orientation and career exploration units at the start of studies, the use of student tutors, problem-based learning, and “polyclinic teaching” in the city’s teaching hospitals. Pioneering new approaches and implementing them in teaching was, even then, not without conflict. Winfried Kahlke was known for his integrity and reliability, yet he could also argue passionately when it came to his convictions and the democratization of medicine. He was deeply engaged with ethical questions in medical education and professional life and instilled this reflective stance in his students, who drew significant inspiration from it in developing their own professional understanding as physicians.

In line with this, Winfried Kahlke founded the long-standing interdisciplinary ethics seminar, which took place monthly during the semester and was – innovatively – open not only to university members but also to other professionals and interested citizens of Hamburg. The great demand for such a forum, in which ethical questions in medicine were discussed at an academic level from multiple perspectives, became quickly evident, and the seminar had a lasting impact well beyond the UKE and into the city.

In addition to his academic work, Winfried Kahlke was deeply committed to medical professional policy. From 1986 to 1994 and again from 1998 to 2002, he served as a board member of the Hamburg medical association. There, he was particularly devoted to continuing medical education, chairing the continuing education committee from 1994 to 2002, for which he was awarded the Ernst von Bergmann Plaque by the German medical association. In 2016, he served as honorary president of the German medical assembly (Deutscher Ärztetag) in Hamburg, and in late April he was awarded the Federal Cross of Merit, First Class.

Winfried Kahlke consistently – also in the face of opposition – stood as a principled advocate of democratic values, minority rights, and human rights, accompanied ethical discourse as a critical thinker, and summarized his life’s approach with the words: “I have always stayed the course.”

Our heartfelt condolences go to his wife Heidi and their four children with their families.

## Competing interests

The author declares that she has no competing interests.

